# Correlative AFM and Scanning Microlens Microscopy for Time‐Efficient Multiscale Imaging

**DOI:** 10.1002/advs.202103902

**Published:** 2022-02-27

**Authors:** Tianyao Zhang, Haibo Yu, Jialin Shi, Xiaoduo Wang, Hao Luo, Daojing Lin, Zhu Liu, Chanmin Su, Yuechao Wang, Lianqing Liu

**Affiliations:** ^1^ State Key Laboratory of Robotics Shenyang Institute of Automation, Chinese Academy of Sciences Shenyang 110016 P. R. China; ^2^ Institutes for Robotics and Intelligent Manufacturing Chinese Academy of Sciences Shenyang 110016 P. R. China; ^3^ University of Chinese Academy of Sciences Beijing 100049 P. R. China

**Keywords:** atomic force microscopy (AFM), correlative microscopy, microlens, microsphere, optical imaging

## Abstract

With the rapid evolution of microelectronics and nanofabrication technologies, the feature sizes of large‐scale integrated circuits continue to move toward the nanoscale. There is a strong need to improve the quality and efficiency of integrated circuit inspection, but it remains a great challenge to provide both rapid imaging and circuit node‐level high‐resolution images simultaneously using a conventional microscope. This paper proposes a nondestructive, high‐throughput, multiscale correlation imaging method that combines atomic force microscopy (AFM) with microlens‐based scanning optical microscopy. In this method, a microlens is coupled to the end of the AFM cantilever and the sample‐facing side of the microlens contains a focused ion beam deposited tip which serves as the AFM scanning probe. The introduction of a microlens improves the imaging resolution of the AFM optical system, providing a 3–4× increase in optical imaging magnification while the scanning imaging throughput is improved ≈8×. The proposed method bridges the resolution gap between traditional optical imaging and AFM, achieves cross‐scale rapid imaging with micrometer to nanometer resolution, and improves the efficiency of AFM‐based large‐scale imaging and detection. Simultaneously, nanoscale‐level correlation between the acquired optical image and structure information is enabled by the method, providing a powerful tool for semiconductor device inspection.

## Graphical abstract

A nondestructive, high‐throughput, multiscale correlation imaging method that combines atomic force microscopy with silica microlens‐based scanning optical microscope is presented. The proposed method effectively bridges the resolution gap between traditional optical imaging and atomic force microscopy (AFM), achieves cross‐scale rapid imaging with micrometer to nanometer resolution, and improves the efficiency of AFM‐based large‐scale imaging and detection.

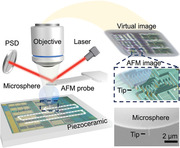

## 1 Introduction

In the field of nanotechnology, the increasing demand for ultra‐efficient and high‐performance microelectronic devices has led to an aggressive reduction of critical feature sizes of ultra and large‐scale integrated circuit devices toward the quantum scale; accordingly, semiconductor fabrication processes are continuously being optimized so that more and more of these devices can be packed into highly complex patterns within a small integrated circuit footprint.^[^ ^1^, ^2^ ^]^ In semiconductor device fabrication, error detection, defect location, and analysis of semiconductor wafers are essential for quality control and process efficiency. Consequently, the reduction in device feature sizes and the increase in integrated circuit pattern complexity require continuous enhancement of the resolution and efficiency of integrated circuit imaging and detection technologies. Thus, a high‐throughput imaging technique that can image large areas quickly and provide circuit node‐level high‐resolution images needs to be developed.

At present, micro‐to‐nanoscale imaging methods include electron microscopy (SEM),^[^ ^3^ ^]^ scanning probe microscopy (SPM),^[^ ^4^, ^5^ ^]^ and optical microscopy (OM). SEM uses a high‐energy electron beam to illuminate the sample, so it has a far higher resolution than OM, which is widely used in semiconductor inspection.^[^ ^6^, ^7^ ^]^ However, in most cases, SEM must be used in a vacuum environment. Furthermore, it is sometimes difficult for SEM to produce a 3D topography of the sample surface.

Since its invention by Binnig et al.,^[^ ^4^ ^]^ atomic force microscopy (AFM) has been widely used in the field of nanotechnology.^[^ ^8^, ^9^, ^10^, ^11^, ^12^, ^13^ ^]^ In addition, the realization of nonmorphological characterization technology is a typical feature of today's AFM. In the case of contact resonance AFM or atomic force acoustic microscopy, certain types of cantilevers could result in an enhancement of the sensitivity, and the mechanical characterization techniques could be easily implemented without any additional requirements.^[^ ^14^ ^]^ The nanometer resolution imaging and high‐sensitivity of AFM make it very useful for the inspection of semiconductor logic nodes. However, the raster‐scanning mode limits the imaging speed and range of AFM significantly.^[^ ^15^, ^16^ ^]^ Moreover, the acquisition time increases significantly if the high‐resolution AFM imaging area of interest is enlarged. To overcome these limitations, it is necessary to combine AFM with auxiliary imaging technologies, such as super‐resolution OM.

Compared with other imaging methods, OM has the advantages of being real‐time, nondestructive, and label‐free, making it effective for high‐throughput and large‐scale imaging. However, the imaging resolution of traditional optical microscopes is limited by the visible light wavelength used to illuminate the sample and the numerical aperture of the system (Abbe's diffraction limit). This physical limit means that the lateral resolution of OM is an order of magnitude lower than that of AFM, and it is very difficult to correlate acquired AFM and optical images directly. Recently, super‐resolution OM, including stochastic optical reconstruction microscopy (STORM),^[^ ^17^ ^]^ photoactivated localization microscopy (PALM),^[^ ^18^ ^]^ and stimulated emission depletion (STED),^[^ ^19^, ^20^ ^]^ has allowed the optical diffraction limit to be exceeded. Although these super‐resolution optical microscopes have significantly improved the optical imaging resolution with some correlation imaging methods being proposed,^[^ ^21^, ^22^, ^23^ ^]^ these technologies are based on fluorescence labeling methods,^[^ ^24^ ^]^ so they are generally limited to life sciences applications rather than to other label‐free, real‐time nanoimaging applications. More recently, coupling dielectric microsphere arrays with traditional optical microscopes has enabled super‐resolution imaging than can resolve 50 nm features under white light illumination by transforming evanescent waves into propagating waves.^[^ ^25^, ^26^ ^]^ In combination with a confocal microscope, the microlens‐based imaging resolution was further improved to 25 nm.^[^ ^27^ ^]^ Imaging technology based on a microsphere lens is applicable to wide range of sample types. Microlens‐based imaging can observe subcellular structures and adenoviruses,^[^ ^28^, ^29^, ^30^ ^]^ as well as perform super‐resolution imaging on nonbiological samples such as semiconductor wafer patterns^[^ ^31^, ^32^, ^33^, ^34^, ^35^ ^]^ and nanostructures.^[^ ^36^, ^37^, ^38^ ^]^ When a single microlens is used for imaging, the imaging field of view (FOV) is limited by the size of the microsphere. To address this issue, microsphere scanning imaging can be achieved through the use of chemical driving,^[^ ^39^ ^]^ acoustic fluids,^[^ ^36^ ^]^ and optical tweezers.^[^ ^40^, ^41^, ^42^ ^]^ In addition, a 3D translation stage can be used to precisely control the microsphere integrated within a conventional microscope objective lens,^[^ ^43^, ^44^ ^]^ a capillary glass tube,^[^ ^45^, ^46^ ^]^ a tungsten probe,^[^ ^47^ ^]^ and an AFM probe. In 2016, Wang et al. proposed microsphere‐based scanning superlens microscopy for the first time and realized non‐invasive super‐resolution imaging over a large area for biological and nonbiological objects.^[^ ^48^ ^]^ This microscopy system can achieve super‐resolution imaging under white light and fluorescence, and bridge the resolution gap between AFM and traditional optical microscopes.^[^ ^48^, ^49^, ^50^ ^]^ The combination of scanning superlens microscopy and AFM has strong potential to address the limitations of AFM.

In this study, we developed a nondestructive, high‐throughput, cross‐scale correlation imaging technology, called correlative AFM and scanning superlens microscopy, for large‐scale optical imaging and high‐resolution sample surface structure information collection. A microlens is coupled with an AFM cantilever with the scanning tip deposited on the microlens surface facing the sample. By combining microlens‐based optical imaging and AFM, the AFM force feedback mechanism can control the spatial position of the microlens during scanning and imaging, and the AFM scanning image can correct the distortion in the microsphere optical image. Three different imaging modes were realized: fast and high‐throughput scanning optical imaging with microlens, AFM imaging of surface fine structure, and microlens‐AFM simultaneous imaging. This method avoids the use of bulky optical systems by introducing a microlens. The experiments demonstrated that correlative AFM and scanning microlens microscopy achieves cross‐scale rapid imaging with micron to nanometer resolution and improves the efficiency of large‐scale imaging and detection based on AFM.

## 2 Results and Discussions

### 2.1 Description of Correlative AFM and Scanning Superlens Microscopy

**Figure 1:**
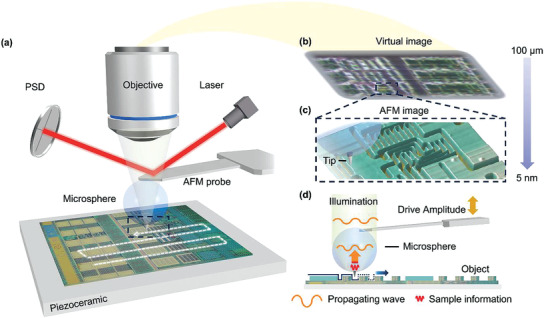
Schematic of correlative AFM and scanning superlens microscopy. a) Overall structure of the correlative AFM and scanning superlens microscopy showing the microlens coupled to the end of the AFM cantilever with the scanning probe tip deposited on the microlens side facing the sample surface. The microlens is integrated with the AFM system to combine AFM imaging with microsphere‐enhanced optical imaging. b) Virtual image formed by scanning superlens microscopy. c) Typical AFM image scanned by the tip deposited under the microlens. d) Scanning and imaging schematic diagram of scanning superlens microscopy.

**Figure** **1**a shows a schematic of the correlative AFM and scanning superlens microscope. In brief, the resolution of the standard AFM optical imaging system is improved by introducing microlens‐based imaging technology into the AFM system. The main components of correlative AFM and scanning superlens microscopy include the AFM system, microlens, and AFM probe tip. The microlens was coupled to the end of the AFM cantilever probe and the scanning probe tip was deposited under the microlens. The optical path of the standard reflective microscope system of the commercial AFM was located above the microlens. In addition, the sample was set on the AFM stage which allows the 3D position of the sample to be controlled. In this study, the resolution of the optical imaging system was enhanced by the coupled microlens, and an enlarged virtual image was formed under the sample plane (Figure 1b). The virtual image can be used for real‐time observation of the characteristic structure of the sample. The spatial position of the microlens above the sample is adjusted by the distance or interaction force control mechanism of the AFM. The scanner and feedback system are used to perform lateral scanning imaging, and its feedback in the vertical direction ensures a constant distance between the microlens and the sample during scanning to maintain stable imaging conditions, as shown in Figure 1d. A camera was used to record the image frame sequence during scanning. Subsequently, the image was cropped and stitched to achieve large‐scale and high‐resolution optical imaging. As shown in Figure 1c, AFM surface imaging and detection can be synchronized with optical imaging by correlative AFM and scanning superlens microscopy, and high‐resolution AFM imaging can be carried out on a selected area after coarse‐level optical imaging.

**Figure 2:**
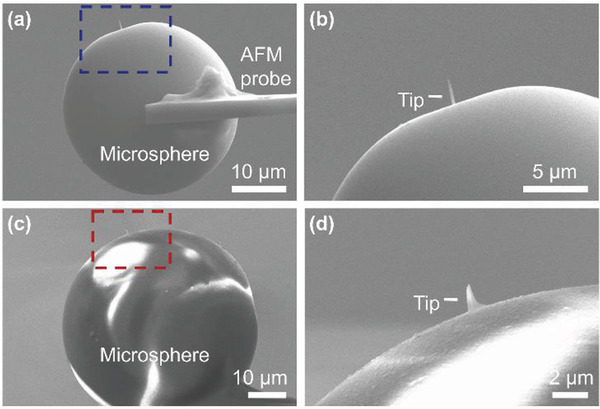
Fabrication and characterization of custom AFM probe with microlens. a) SEM image of the 35 µm diameter microlens installed at the end of the AFM cantilever with the probe tip deposited on the microlens. b) Expanded view of (a). The height of the deposited tip is ≈1.7 µm. c) SEM image of the 50 µm diameter microlens. d) Expanded view of (c). The height of the deposited tip is ≈1.2 µm.

To integrate the microlens into the atomic force microscope system, a custom AFM probe coupled with a microlens was fabricated. This is a new class of end effector that we call a microlens‐AFM probe. The diameter of the attached microsphere determines the imaging FOV and resolution. As shown in **Figure** **2**, silica microspheres with a diameter of 25–60 µm were attached to the end of the AFM cantilever using ultraviolet‐curable glue. The placement of the silica microspheres on the edge of the AFM cantilever was carefully controlled to ensure that the microsphere area covered by the cantilever and glue is less than 20%. This ensures that the cantilever does not interfere with optical path and affect the optical imaging performance of the microlens (preparation details are provided in the Experimental Section). Focused ion beam (FIB) deposition was then used to create a diamond tip on the surface of the microlens coupled to the AFM probe. The height, diameter, and position of the tip were carefully designed (see Figure S1, Supporting Information) to ensure that the distance between the microlens and the sample was within the microsphere‐enhanced optical imaging working distance and so that the tip of the custom microlens‐AFM probe provides good AFM imaging performance. Figure 2 shows representative SEM images of the fabricated microlens‐AFM probes. Figure 2a,b shows a microlens‐AFM probe with a 35 µm diameter microlens deposited with a 1.7 µm tip while Figure 2c,d shows 50 µm diameter microlens with a 1.2 µm tip.

### 2.2 Microlens‐AFM Probe Parameter Analysis and AFM Scanning Imaging

**Figure 3:**
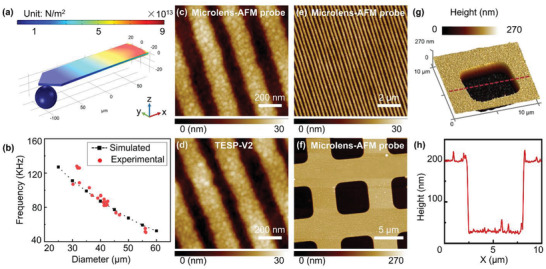
Experimental AFM imaging performance of the custom microlens‐AFM probe. a) The finite‐element model of the microlens‐AFM probe used for numerical stress analysis. b) The characteristic frequency of the custom microlens‐AFM probe coupled with different diameter microlenses; the experimental results are essentially consistent with the simulation results. c) AFM tapping scanning mode image of a Blu‐ray disk (BD) surface imaged using the microlens‐AFM probe. d) AFM scanning image of a BD surface using a commercial AFM probe. e) Large‐area AFM scanning image using the microlens‐AFM probe. f) AFM scanning image of a standard silicon calibration sample using the microlens‐AFM probe. g) 3D view of the standard silicon calibration sample. h) AFM scanned profiles corresponding to the cross sections in (g).

The microlens‐AFM probe was characterized so that the appropriate scanning parameters for prolonging the lifespan of the AFM probe tip and maintaining the near‐field imaging capability of the microlens could be determined. We used tapping mode AFM on the sample surface to analyze the imaging performance of the deposited tip. In this study, a commercial AFM (Dimension Icon, Bruker) was used, and the standard AFM probes were replaced with our custom microlens‐AFM probes. First, AFM scanning imaging parameters need to be properly configured experimentally and theoretically. To numerically analyze the mechanical characteristics of the microlens‐AFM probe, a finite‐element theoretical model was constructed using COMSOL Multiphysics (**Figure** **3**a). As shown in Figure 3b, the natural inherent frequency of the microlens‐AFM probe decreases with an increase in the diameter of the microlens, and the results are consistent with the resonant frequency of the microlens‐AFM probe in the experiment (Figure S2, Supporting Information). In addition, the characteristic frequency is dependent on the mechanical properties of the individual AFM probe, and the position of the microlens on the AFM cantilever must be precisely controlled. The microlens size and the AFM cantilever spring constant should be selected to ensure high‐quality AFM imaging and extend the probe life. A commercially available AFM probe (TESP probe, Bruker, USA) was used for experimental verification and it was determined that the microlens diameter should be less than 80 µm. Figure 3c,e shows the AFM imaging results of the 210 nm interval 110 nm stripe structure of a Blu‐ray disk (BD) surface using a microlens‐AFM probe with a 43 µm diameter silica microsphere coupled to a TESP AFM cantilever probe. The free amplitude of the custom microlens‐AFM probe was determined to be 53.4 nm. The probe drive amplitude was configured to be 39.4 nm and the frequency was 94.0 kHz. Subsequently, in the process of scanning images, the amplitude setpoint was adjusted until the two scan lines of trace and retrace coincide so as to optimize the amplitude setpoint, with the value set at 11.7 nm.^[^ ^51^, ^52^ ^]^ Images of the samples were taken in tapping mode with a horizontal scan rate of 0.5 Hz. The results show that the AFM imaging range is 2 or 10 µm, and in other areas (Figure S3, Supporting Information), the imaging effect of AFM is comparable to that of commercial probes (Figure 3d) with a 13.5 nm drive amplitude, 285.5 kHz drive frequency, and 1.6 nm amplitude setpoint. Furthermore, to evaluate the performance of these probes in imaging large‐scale structures, a standard silicon calibration sample with 5 µm square features 160 nm deep were also imaged using a custom microlens‐AFM probe with a 50 µm diameter silica microsphere (Figure 3f,g). We analyzed the AFM scanned profiles (Figure 3h) corresponding to the cross sections marked with red dotted lines in Figure 3g. As revealed by the resulting AFM images, the custom microlens‐AFM probe is sufficiently accurate to follow the tall structures and steep curvature of the sample, without crashing or sticking.

**Figure 4:**
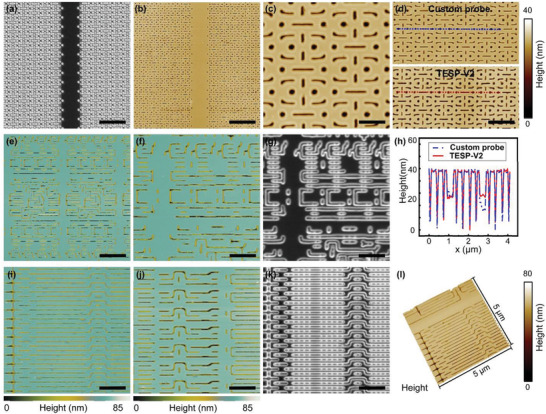
Semiconductor wafer surface structure imaging with a microlens‐AFM probe in tapping scanning mode. a) SEM image of a semiconductor wafer surface containing point and line patterns with a minimum feature size of 50 nm. b,c) AFM image of the semiconductor wafer pattern with microlens‐AFM probe using tapping scanning mode. d) AFM images of the same wafer pattern using a microlens‐AFM probe (top) and a commercial AFM probe (bottom). e,f) AFM image of the 60 nm pitch semiconductor wafer pattern using a microlens‐AFM probe. g) SEM image of the 60 nm pitch semiconductor wafer pattern. h) AFM line profiles corresponding to the cross sections in (d). i,j) AFM image of the 70 nm pitch semiconductor wafer pattern using a microlens‐AFM probe. k) SEM image of the semiconductor wafer pattern corresponding to (i). l) 3D view of the semiconductor wafer pattern. Scale bars, 2 µm (a, b, e); 1 µm (d, f, g, i, j, k); 400 nm (c).

To further evaluate the scanning imaging performance of the microlens‐AFM probe, we performed AFM imaging of a semiconductor wafer containing parallel line patterns with a pitch distance of 70 nm (**Figure** **4**k), intricate patterns with 60 nm features (Figure 4g), and point and line patterns with a 50 nm pitch (Figure 4a). We characterized the microlens‐AFM probe with a 60 µm diameter microsphere (measured resonant frequency ≈ 50.2 kHz) by scanning the structures using tapping mode at a scan rate of 0.3 Hz. Figure 4b–d shows unfiltered AFM images of the wafer surface with a minimum feature size of 50 nm. The microlens‐AFM probe accurately tracked the fine structure of the 20 nm high point and line patterns with very little image distortion. Comparing the two images acquired from the same sample area using a commercial probe and a microlens‐AFM probe reveals that the measured size, shape, and height of the patterns and the pitch spacing were nearly identical (Figure 4d), and the line profiles corresponding to the cross‐sections overlap (Figure 4h). In addition, we scanned semiconductor wafer patterns with different pitches and different scanning areas using a microlens‐AFM probe. The results show that the new probe can achieve accurate large‐area image scanning at high‐speed (Figure S4, Supporting Information). The durability of the probe was verified (Figure S5, Supporting Information). After 6 h of imaging, SEM examination of the tip revealed no appreciable damage to the tip.

### 2.3 Optical Imaging of Scanning Microlens

**Figure 5:**
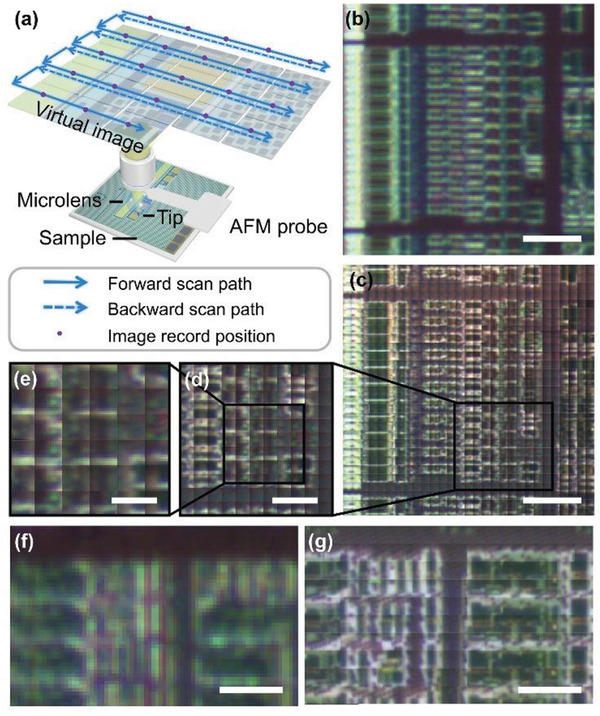
Optical imaging with the scanning microlens. a) Schematic of the AFM‐microlens scanning imaging system. b,f) Image directly observed using the standard AFM optical system. c,g) Semiconductor wafer surface imaged using scanning superlens microscopy. d) Expanded view of (c). e) Expanded view of (d). Scale bars, 20 µm (b, c); 10 µm (d, f, g); 5 µm (e).

The scanning superlens probe improved the large‐scale optical imaging resolution of the standard optical microscope (2 µm imaging resolution) of the AFM system. A schematic of the microlens scanning system is shown in **Figure** **5**a. The reflective optical system of the AFM consisted of a 10× objective lens, a 5‐megapixel camera (2560 × 1920), and a light source located above the microlens. In the experiment, silica microspheres with a diameter of 35 µm and a refractive index of 1.46 were used as microlenses to image the semiconductor wafer surface (Figure 5b). Microlens imaging was combined with the standard AFM optical microscope system to obtain a magnified virtual image in air and improve the imaging resolution. It is worth noting that the imaging plane is below the sample surface, so the image observed through the microlens is a virtual image. The focal length and imaging plane position of 30–60 µm diameter microlenses were analyzed through finite‐difference time‐domain (FDTD) simulations and experimental observations. To verify the optical imaging performance of the microlens, a semiconductor wafer stripe structure composed of 220 nm‐wide lines 1 µm apart, which could not be discerned by the standard AFM optical system (Figures S6–S8, Supporting Information), was imaged. An optical microscope image of the stripe structure enhanced by a 43 µm diameter microlens is shown in Figure S6c (Supporting Information). With the aid of the microlens, the stripe was magnified with a magnification factor of 3.11×. To further analyze the imaging characteristics of the microlens, the structural size data of samples (stripe structure with 220‐nm‐wide lines separated by 1 µm) measured by SEM was considered as the standard, a 1D rectangular function set and convoluted with the Gaussian fitting point spread function, and then matched with the experimental data observed by the microlens, so as to calculate the point spread function of the system (PSF). We estimated that the imaging resolution of the scanning microlens system is ≈550 nm with the silica microsphere (Figure S7, Supporting Information). We then studied the imaging depth via the influence of the distance between the microlens and the sample (∆*z*) on the imaging performance. The clearest images observed by the microlens at different ∆*z* were recorded, as shown in Figure S8 (Supporting Information). Evidently, when ∆*z* is less than 10 µm, the microlens can maintain the imaging quality of the semiconductor wafer sample, and the distance has little effect on the results. Therefore, we speculate that in the tapping mode, the deposition tip height of the custom microlens‐AFM probes is less than 10 µm, which can ensure the optical imaging of the microlens within the appropriate working distance.

The scanning system of the AFM was used to perform lateral scanning imaging. The AFM force feedback mechanism controls the spatial position of the microlens during imaging and maintains stable imaging conditions during the scanning process. The AFM optical system camera was used to record the image sequence during scanning. Then, the recorded images were cropped and stitched to achieve large‐scale, high‐resolution optical images (Figure 5c,g). Compared with the imaging results of the AFM optical system without microlens assistance, the imaging resolution is significantly improved by microlens imaging, and structures with ≈300 nm features can be resolved; this can aid the coarse detection and positioning of the semiconductor wafer. The total scanning area was 90 × 90 µm^2^ (Figure 5c). For this area, commercial AFM scanning requires at least 256 scanning lines, and to ensure high‐resolution imaging the actual acquisition time is 40 min at a scanning rate of 0.1 Hz. However, with microlens‐assisted scanning imaging, only 32 lines need to be scanned, and the acquisition time is reduced significantly to 5 min. Compared with traditional AFM, the imaging efficiency is improved ≈8×, and this efficiency is dependent on the camera frame rate. Using a camera with a higher frame rate can further shorten the imaging time.

### 2.4 Correlative AFM and Scanning Superlens Microscopy

**Figure 6:**
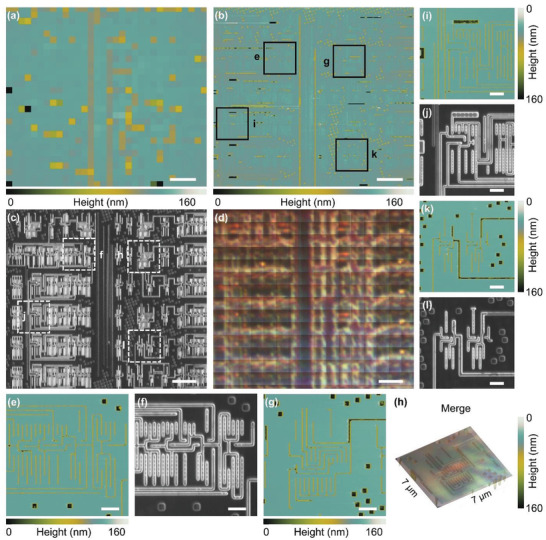
Semiconductor wafer surface imaging by correlative AFM and scanning superlens microscopy. a) AFM image of the semiconductor wafer surface from high‐throughput 32‐line scanning. b) AFM image of semiconductor wafer surface. c) SEM image. d) Semiconductor wafer subsurface structures imaged through 32‐line scanning optical imaging with microlens enhancement. f,j,l) SEM images of semiconductor wafer surface fine structure. e,g,i,k) AFM images of semiconductor wafer surface fine structure. h) Correlative AFM/scanning microlens microscopy image of semiconductor wafer surface. Scale bars, 5 µm (a–d); 1 µm (e–g, i–l).

The combination of AFM and microlens‐enhanced optical imaging technology can provide both fast imaging and node‐level high‐resolution imaging, thus improving the efficiency of AFM‐based imaging and detection. Consequently, the requirements of high‐throughput and large‐scale imaging in semiconductor foundries can be achieved. **Figure** **6** shows the scanning imaging results of special features on the semiconductor wafer surface under different imaging modes using correlative AFM and scanning superlens microscopy. The sample surface was scanned in air at a scan rate of 0.2 Hz using the customized probe with a 50‐µm diameter silica microlens and a 2 µm long diamond probe tip. The special structures on the semiconductor wafer were inspected using three different imaging modes: fast and high‐throughput scanning optical imaging with microlens, AFM imaging of surface fine structure, and microlens‐AFM simultaneous imaging (Movie S1, Supporting Information). The interface of the microlens and AFM scanning imaging simultaneously of the periodic stripe structure on the semiconductor wafer surface is shown in Movie S2 (Supporting Information). Figure 6a shows the AFM image of the semiconductor wafer surface acquired by 32 lines of fast and high‐throughput scanning, with a total scanning area of 40 × 40 µm^2^. The optical images recorded by scanning superlens microscopy were cropped and stitched to obtain large‐area imaging results (Figure 6d). Comparing the optical image and the AFM image obtained simultaneously by the microlens high‐throughput scanning optical imaging mode, it can be observed that under the same conditions, the microlens scanning optical imaging obtains more sample information. Standard AFM imaging needs to scan at least 256 lines at a scanning speed of 0.2 Hz to ensure image quality (Figure 6b). However, scanning optical imaging only requires 32 lines of scanning, that is, the throughput is increased 8×, with an acquisition time of ≈2.5 min. In addition, optical information that are not present in the SEM (Figure 6c) and AFM images (Figure 6b) were acquired. A video of the scanning process corresponding to the result shown in Figure 6d,k is provided in Movie S1 (Supporting Information). After the coarse detection of the chip, high‐resolution imaging of specific areas with nanometer‐scale features was carried out using the AFM imaging mode of the correlative imaging system (Figure 6e,g,i,k). In this mode, the optical image can also be obtained simultaneously, merge the optical image with AFM scans as shown in Figure 6h. Here, we introduce microlens scanning imaging as an intermediate semiconductor inspection method. High‐throughput large‐scale optical detection can be carried out on the semiconductor wafer before fine imaging by AFM. The analysis of the optical imaging results can identify target areas for fine AFM imaging. This correlative AFM and scanning superlens imaging method enables efficient semiconductor chip inspection, as shown in Figure 6 and Figure S9 (Supporting Information).

### 2.5 Numerical Simulation and Analysis of Optical Imaging Characteristics

**Figure 7:**
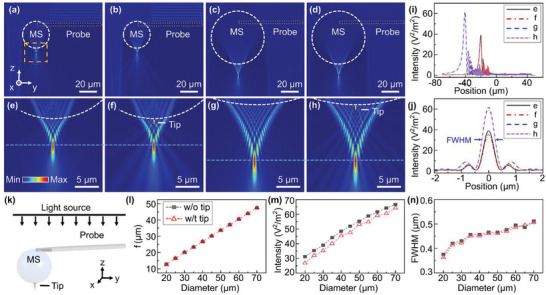
Numerical simulation results of the optical intensity distributions. FDTD simulation results for microlens‐AFM probe. a,c) Without and b,d) with tip deposited, the microsphere diameters are 30 µm (a,b) and 60 µm (c,d). e–h) Expanded view of (a–d). i) Field‐intensity distributions along the *z*‐axis of the central axis of the microspheres in (e–h). j) Intensity distribution along the *y*‐axis at the maximum intensity position of PNJs. k) Microlens‐AFM probe with plane wave illumination. Simulated results show the deposited tip affected the focusing properties, l) focus position, m) FWHM, and n) the PNJ intensity.

The optical imaging performance of microlenses is fundamentally related to its focusing properties.^[^ ^25^ ^]^ Previous studies have confirmed that the probe‐combined microlens has little influence on the optical properties of microlenses. To numerically analyze the deposited tip‐induced effects on the focusing properties of the microlens, a theoretical finite‐element model was constructed and analyzed by FDTD simulations and the results are shown in **Figure** **7**. In the simulation, a plane wave with a peak wavelength of *λ* = 550 nm incident along the *z*‐axis radiated onto the microlens‐AFM probe immersed in air (refractive index, n1 = 1). The refractive index of the silica microsphere is n2 = 1.5 and refractive index of the deposited diamond tip is n3 = 2.4 (Figure 7k).

The incident plane wave is highly focused on a small area by the microspheres, resulting in the formation of a photonic nanojet (PNJ) on its shadow surface side. Figure 7a–j shows the FDTD simulated field‐intensity distributions along the *y* and *z* planes of the microlens‐AFM probe with and without the tip deposited for the 30 and 60 µm diameter microspheres. It can be seen that the diamond tip causes local field interference, but the microlens‐AFM probe still produces a PNJ similar to that without the deposited tip. Figure 7l–n summarizes the quantitative simulation results and show the focusing characteristics of the microspheres: focus position (f), full‐width‐at‐half‐maximum (FWHM), and the PNJ intensities of microspheres affected by the deposited tip and the diameter of the microspheres. The PNJ intensity was slightly changed by the presence of the tip, but the focus position and FWHM did not change significantly. The deposited tip induced a gradual decrease in the PNJ intensity with an increase in the microsphere diameter, which is consistent with the experimental results. In conclusion, the combined microlens‐AFM probe does not cause significant changes on the focusing characteristics of the microsphere, and the probe tip has a very minor effect on the near‐field optical properties.

## 3 Conclusion 

In summary, we demonstrated a nondestructive, high‐throughput, cross‐scale correlation imaging technology, namely, correlative AFM and scanning superlens microscopy, for large‐scale optical imaging and rich sample surface structure information acquisition. By coupling the microlens with an AFM probe and depositing a scanning tip on the microlens surface facing the sample, microlens‐based optical imaging and AFM can be combined and the AFM force feedback mechanism can enable accurate control of the spatial position of the microlens during scanning and imaging. Three imaging modes are realized: fast and high‐throughput scanning optical imaging with microlens, AFM imaging of surface fine structure, and microlens‐AFM simultaneous imaging. Experimental results verified that the introduction of a microlens improves the imaging resolution of the conventional AFM optical system with a 3–4× increase in imaging magnification, effectively bridging the resolution gap between traditional optical imaging and AFM. The imaging throughput is improved ≈8× compared to a commercial AFM. High‐throughput and high‐resolution correlative AFM and scanning superlens microscopy achieves cross‐scale rapid imaging with micrometer to nanometer resolution and improves the efficiency of AFM‐based large‐scale imaging and detection. The imaging efficiency in the scanning optical imaging mode is mainly limited by the scanning speed of the system and the frame rate of the camera. Therefore, improving the scanning speed and using high‐speed cameras will further improve the imaging throughput. Moreover, the nanoscale correlation of the two imaging products can provide valuable supplementary information from a single scan, enabling real‐time optical imaging distortion correction and providing optical surface morphology information on the sample area being measured by AFM. In addition, the custom microlens‐AFM probe designed and fabricated in this study can be used to expand the capabilities of traditional microscopes and can be used with commercial AFM scanning systems with the advantages of high efficiency, flexibility, and portability. Correlative AFM and scanning superlens microscopy also enables in situ time‐efficient multiscale observation with high resolution and can potentially be applied for imaging biological samples.

## 4 Experimental Section

### Experimental System and Imaging Equipment

Correlative microscopy images were acquired with a commercial AFM microscope (Dimension Icon, Bruker, USA) equipped with a standard probe holder. Custom microlens‐AFM probes with a nominal spring constant of 42 N m^−1^ and a resonant frequency of 50–120 kHz were used to image the semiconductor wafer samples. The spatial position of the microlens above the sample was adjusted by the interaction force control mechanism of the AFM. The scanner and feedback system enable lateral scanning imaging, and feedback in the vertical direction ensures a constant distance between the microlens and the sample in the scanning process to maintain stable imaging conditions. The tapping mode was used to perform AFM scanning on the sample surface in air at a scan rate of 0.1–0.8 Hz with 1–90 µm square imaging areas. Optical images were captured by the AFM optical system camera at a resolution of 640 × 480 and a frame rate of 30 Hz. SEM images were obtained using a Zeiss EVO MA10.

### Numerical Simulation and Analysis

FDTD simulation software (Lumerical, Ansys Canada Ltd., Vancouver, Canada) was used to perform numerical analyses of the field‐intensity distribution and study the influence of the tip deposited on the microsphere surface on the optical focusing properties. In this simulation, a silica microsphere (refractive index n1 = 1.5) in air (refractive index n2 = 1) was used, and the relative refractive index was ≈1.5; the incident light was a plane wave with a peak wavelength of *λ* = 550 nm, incident along the *z*‐axis. The probe model was created based on the real size of the cantilever of the AFM probe. Finally, the optical field‐intensity distributions and PNJ properties were analyzed through simulations.

### Fabrication of the Custom Microlens‐AFM Probe

Silica microspheres (6‐7‐1000, BaseLine, China) 25–60 µm in diameter with a refractive index of 1.46 were used as microlenses for imaging in air. Using an optical microscope and a precision translation stage, the microlens was attached to the edge of the AFM probe end using ultraviolet‐curable glue (NOA63, Edmund Optics). The area covered by the cantilever and glue on the microsphere should be less than 20% of the surface of the microsphere. This ensures that the cantilever does not interfere with the optical path and affect the optical imaging performance of the microlens. The ultraviolet‐curable glue was cured with 100 W irradiation for 30 min using a halogen lamp after being injected, then the glue was injected again between the microlens and the AFM probe and cured for a further 40 min, so that the microspheres could be stably coupled to the AFM probe. FIB deposition was used to create a diamond tip on the surface of the microlens. The length of the tip was designed according to the diameter of the microlens to ensure that the distance between the microlens and the sample was within the microsphere‐enhanced optical imaging working distance. The diameter of the tip was 200–500 nm for the base and the tip radius was less than 20 nm, and the deposited tip was perpendicular to the sample surface to realize different functions, such as AFM scanning imaging, nanomanipulation, and nanomanufacturing on the sample surface. Prior to tip deposition by FIB, a gold film with a thickness of ≈5 nm was sputtered on the surface of the microspheres. After tip deposition the gold film was dissolved by dipping the microsphere in I‐KI solution for 3–5 min followed by rinsing with deionized water.

### Statistical Analysis

AFM images were analyzed using Nanoscope Analysis 1.9 (Figures 3, 4, and 6; Figures S3 and S4, Supporting Information). Statistical analysis of the average characteristic sizes of sample structures were manually counted and the sample size for each condition was 20, the values represented the mean (Figures 3 and 4; Figure S6, Supporting Information). The optical image observed by the microlens was fitted and determined by the Gaussian fitting point spread function written by MATLAB 2018 (Figure S7, Supporting Information). The intensity of contour information extracted from optical imaging results was normalized preprocessing (Figures S6–S8, Supporting Information).

## Conflict of Interest

The authors declare no conflict of interest.

## Author Contributions

The manuscript was written through contributions of all authors. All authors have given approval to the final version of the manuscript.

## Supporting information

Supporting Information

Supplemental Movie 1

Supplemental Movie 2

## Data Availability

The data that support the findings of this study are available from the corresponding author upon reasonable request.

## References

[1] N. Beginning , Comput. Sci. Eng. 1975, 19, 41.

[2] K. Agarwal , R. Chen , L. S. Koh , C. J. R. Sheppard , X. Chen , Phys. Rev. 2015, 5, 021014.

[3] D. Mcmullan , Scanning 1995, 17, 175.

[4] G. Binnig , C. F. Quate , C. Gerber , Phys. Rev. Lett. 1986, 56, 930. 10033323 10.1103/PhysRevLett.56.930

[5] B. Drake , C. B. Prater , A. L. Weisenhorn , S. A. C. Gould , T. R. Albrecht , C. F. Quate , D. S. Cannell , H. G. Hansma , P. K. Hansma , Science 1989, 243, 1586. 2928794 10.1126/science.2928794

[6] A. Vaid , B. Bin Yan , Y. T. Jiang , M. Kelling , C. Hartig , J. Allgair , P. Ebersbach , M. Sendelbach , N. Rana , A. Katnani , E. Mclellan , C. Archie , C. Bozdog , H. Kim , M. Sendler , S. Ng , B. Sherman , B. Brill , I. Turovets , R. Urensky , Metrol. Insp. Process Control Microlithogr. 2011, 7971, 797103.

[7] T. F. Yao , A. Duenner , M. Cullinan , Precis. Eng. 2017, 47, 147.

[8] Y. Kim , C. M. Lieber , Science 1992, 257, 375. 17832835 10.1126/science.257.5068.375

[9] Y. Sugimoto , P. Pou , O. Custance , P. Jelinek , M. Abe , R. Perez , S. Morita , Science 2008, 322, 413. 18927388 10.1126/science.1160601

[10] S. Kim , D. C. Ratchford , X. Li , ACS Nano 2009, 3, 2989. 19751065 10.1021/nn900606s

[11] Y. Wen , H. Lu , Y. Shen , H. Xie , IEEE Trans. Ind. Electron. 2019, 67, 2916.

[12] H. Xie , D. S. Haliyo , S. Régnier , Appl. Phys. Lett. 2009, 94, 153106.

[13] Y. Shen , M. Nakajima , Z. Zhang , T. Fukuda , IEEE/ASME Trans. Mechatronics 2015, 20, 3009.

[14] M. Muraoka , Nanotechnology 2005, 16, 542.

[15] Y. F. Dufrêne , T. Ando , R. Garcia , D. Alsteens , D. Martinez‐martin , A. Engel , C. Gerber , D. J. Müller , Nat. Publ. Gr. 2017, 12, 295. 10.1038/nnano.2017.45 28383040

[16] B. P. Brown , L. Picco , M. J. Miles , C. Faul , Small 2013, 9, 3201. 23609982 10.1002/smll.201203223

[17] M. J. Rust , M. Bates , X. Zhuang , Nat. Methods 2006, 3, 793. 16896339 10.1038/nmeth929 PMC2700296

[18] E. Betzig , G. H. Patterson , R. Sougrat , O. W. Lindwasser , S. Olenych , J. S. Bonifacino , M. W. Davidson , J. Lippincott‐Schwartz , H. F. Hess , Science 2006, 313, 1642. 16902090 10.1126/science.1127344

[19] S. W. Hell , J. Wichmann , Opt. Lett. 1994, 19, 780. 19844443 10.1364/ol.19.000780

[20] T. A. Klar , S. W. Hell , Opt. Lett. 1999, 24, 954. 18073907 10.1364/ol.24.000954

[21] P. D. Odermatt , A. Shivanandan , H. Deschout , R. Jankele , A. P. Nievergelt , L. Feletti , M. W. Davidson , A. Radenovic , G. E. Fantner , Nano Lett. 2015, 15, 4896. 26121585 10.1021/acs.nanolett.5b00572

[22] A. I. Gómez‐Varela , D. R. Stamov , A. Miranda , R. Alves , C. Barata‐Antunes , D. Dambournet , D. G. Drubin , S. Paiva , P. A. A. De Beule , Sci. Rep. 2020, 10, 1122. 31980680 10.1038/s41598-020-57885-z PMC6981207

[23] B. Harke , J. V. Chacko , H. Haschke , C. Canale , A. Diaspro , Opt. Nanoscopy 2012, 1, 3.

[24] S. El‐Kirat‐Chatel , Y. F. Dufreîne , ACS Nano 2012, 6, 10792. 23146149 10.1021/nn304116f

[25] J. Y. Lee , B. H. Hong , W. Y. Kim , S. K. Min , Y. Kim , M. V. Jouravlev , R. Bose , K. S. Kim , I.‐C. Hwang , L. J. Kaufman , C. W. Wong , P. Kim , K. S. Kim , Nature 2009, 460, 498.

[26] Z. Wang , W. Guo , L. Li , B. Luk’Yanchuk , A. Khan , Z. Liu , Z. Chen , M. Hong , Nat. Commun. 2011, 2, 218. 21364557 10.1038/ncomms1211

[27] Y. Yan , L. Li , C. Feng , W. Guo , S. Lee , M. Hong , ACS Nano 2014, 8, 1809. 24471860 10.1021/nn406201q

[28] H. Yang , N. Moullan , J. Auwerx , M. A. M. Gijs , Small 2014, 10, 1712. 24914446 10.1002/smll.201302942

[29] A. Darafsheh , C. Guardiola , A. Palovcak , J. C. Finlay , A. Cárabe , Opt. Lett. 2015, 40, 5. 25531594 10.1364/OL.40.000005

[30] L. Li , W. Guo , Y. Yan , S. Lee , T. Wang , Light: Sci. Appl. 2013, 2, e104.

[31] W. Fan , B. Yan , Z. Wang , L. Wu , Sci. Adv. 2016, 2, 40. 10.1126/sciadv.1600901 PMC4982708 27536727

[32] F. Wang , L. Liu , P. Yu , Z. Liu , H. Yu , Y. Wang , W. J. Li , Sci. Rep. 2016, 6, 24703. 27102207 10.1038/srep24703 PMC4840372

[33] H. Yang , M. A. M. Gijs , Microelectron. Eng. 2015, 143, 86.

[34] X. Hao , C. Kuang , X. Liu , H. Zhang , Y. Li , Appl. Phys. Lett. 2011, 99, 2012.

[35] A. Darafsheh , G. F. Walsh , L. Dal Negro , V. N. Astratov , Appl. Phys. Lett. 2012, 101, 2010. 10.1063/1.3684246 PMC3293362 22396621

[36] G. Jin , H. Bachman , T. D. Naquin , J. Rufo , S. Hou , Z. Tian , C. Zhao , T. J. Huang , ACS Nano 2020, 14, 8624. 32574033 10.1021/acsnano.0c03009 PMC7438315

[37] B. Jia , F. Wang , H. Chan , G. Zhang , W. J. Li , Microsystems Nanoeng. 2019, 5, 1. 10.1038/s41378-018-0040-3 PMC6330505 31057928

[38] A. Darafsheh , N. I. Limberopoulos , J. S. Derov , D. E. Walker , V. N. Astratov , Appl. Phys. Lett. 2014, 104, 061117.

[39] J. Li , W. Liu , T. Li , I. Rozen , J. Zhao , B. Bahari , B. Kante , J. Wang , Nano Lett. 2016, 16, 6604. 27608508 10.1021/acs.nanolett.6b03303

[40] Y. Li , X. Liu , B. Li , Light: Sci. Appl. 2019, 8, 61. 31645911 10.1038/s41377-019-0168-4 PMC6804537

[41] X. Chen , T. Wu , Z. Gong , Y. Li , Y. Zhang , B. Li , Photonics Res 2020, 8, 225.

[42] Y. Li , H. Xin , X. Liu , Y. Zhang , H. Lei , B. Li , ACS Nano 2016, 10, 5800. 27163754 10.1021/acsnano.5b08081

[43] B. Yan , Z. Wang , A. L. Parker , Y. Lai , P. J. Thomas , L. Yue , J. N. Monks , Appl. Opt. 2017, 56, 3142. 28414373 10.1364/AO.56.003142

[44] B. Yan , Y. Song , X. Yang , D. Xiong , Z. Wang , Appl. Opt. 2020, 59, 2641. 32225809 10.1364/AO.386504

[45] M. Michihata , K. Takami , T. Hayashi , Y. Takaya , Adv. Opt. Technol. 2014, 3, 417.

[46] L. A. Krivitsky , J. J. Wang , Z. Wang , B. Luk'yanchuk , Sci. Rep. 2013, 3, 3501. 24336231 10.1038/srep03501 PMC3863983

[47] Y. Wen , H. Yu , W. Zhao , F. Wang , X. Wang , L. Liu , W. J. Li , IEEE Trans. Nanotechnol. 2019, 18, 226.

[48] F. Wang , L. Liu , H. Yu , Y. Wen , P. Yu , Z. Liu , Y. Wang , W. J. Li , Nat. Commun. 2016, 7, 13748. 27934860 10.1038/ncomms13748 PMC5476830

[49] T. Zhang , H. Yu , P. Li , X. Wang , F. Wang , J. Shi , Z. Liu , P. Yu , W. Yang , Y. Wang , L. Liu , ACS Appl. Mater. Interfaces 2020, 12, 48093. 32960563 10.1021/acsami.0c12126

[50] S. Wang , D. Zhang , H. Zhang , X. Han , R. Xu , Microsc. Res. Tech. 2015, 78, 1128. 26515760 10.1002/jemt.22595

[51] R. Garcia , A. S. Paulo , Phys. Rev. B 1999, 60, 4961.

[52] Á. S. Paulo , R. García , Phys. Rev. B 2002, 66, 041406.

