# Supplementary material for: Correlative AFM and Scanning Microlens Microscopy for Time‐Efficient Multiscale Imaging
Source: Adv Sci (Weinh). 2022 Feb 27;9(12):2103902. doi: 10.1002/advs.202103902 (PMC9036010; doi:10.1002/advs.202103902)
Supplement: Supplementary file 1 — Supporting Information [file ADVS-9-2103902-s002.pdf]

Supporting Information

Correlative AFM and scanning microlens microscopy for time-efficient multiscale imaging

Tianyao Zhang[1, 2, 3], Haibo Yu[1, 2, *], Jialin Shi[1, 2], Xiaoduo Wang[1, 2], Hao Luo[1, 2, 3], Daojing Lin[1, 2, 3], Zhu Liu[1, 2], Chanmin Su[1, 2], Yuechao Wang[1, 2], and Lianqing Liu[1, 2, *]

1. State Key Laboratory of Robotics, Shenyang Institute of Automation, Chinese Academy of Sciences, Shenyang 110016, China
2. Institutes for Robotics and Intelligent Manufacturing, Chinese Academy of Sciences, Shenyang 110016, China
3. University of Chinese Academy of Sciences, Beijing 100049, China

* Corresponding authors

To whom correspondence should be addressed. E-mail: yuhaibo@sia.cn; lqliu@sia.cn

Fabrication and characterization of custom AFM probe with microlens

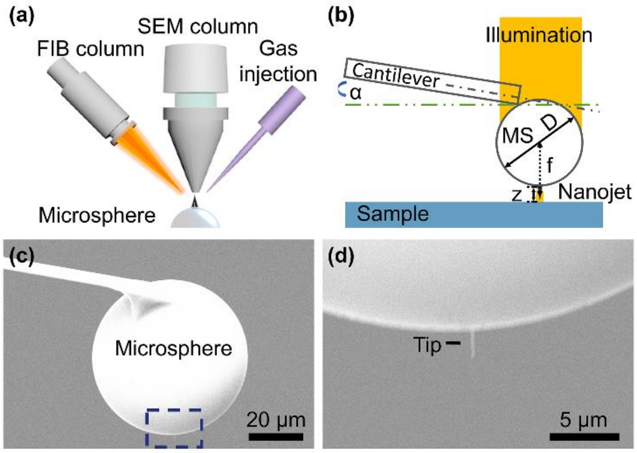

Figure S1. Fabrication and characterization of custom AFM probe with microlens. (a) Schematic diagram of deposition a diamond tip on the back of the microlens coupled to the AFM probe using FIB technology. (b) Design and calculation of the height, diameter and position of the tip. (c) SEM image of the microlens with combined at the end of the AFM cantilever and the probe tip is deposited on the microlens. (d) Expanded view of (c).

First, a silica microsphere with a diameter of 25-60 μm is adhered to the end of the AFM probe through using ultraviolet-curable glue. After the ultraviolet-curable glue was cured by a halogen lamp the microsphere could be stably coupled to the AFM probe. Subsequently, deposit a diamond tip on the back of the microlens coupled to the AFM probe using FIB technology (Figure S1a). The length of the tip is designed according to the diameter of the microlens to ensure that the distance between the microlens and the sample is within the microsphere-enhanced optical imaging working distance (Figure S1b). The fabricated microlens-AFM probe is shown in Figure S1c, the diameter of the microlens is 57 μm, the length of deposition tip is 1.7 μm and the diameter is 220 nm for the base and the tip radius less than 20 nm.

Tuning the driving frequency and amplitude of the microlens-AFM probe

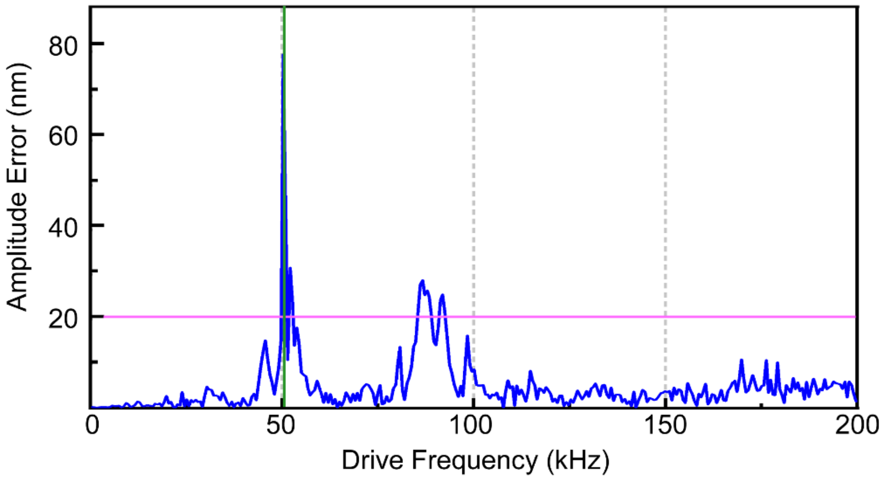

Figure S2. Tuning the driving frequency and amplitude of the microlens-AFM probe in tapping mode.

In the experiment, the resonance peak of the probe needs to be tuned to find the natural inherent frequency of the probe in tapping mode. Figure S2 illustrates the driving frequency and amplitude of the microlens-AFM probe with a 60 μm diameter silica microsphere combined on the TESP probe cantilever. Configure the probe drive amplitude to be 83.2 nm, and the frequency is 50.2 kHz as shown in Figure S2. Subsequently, in the process of scanning images, adjust the amplitude setpoint until the two scan lines of trace and retrace are basically the same to optimize the setpoint as 19.6 nm.

Experimental AFM imaging results of the custom microlens-AFM probe

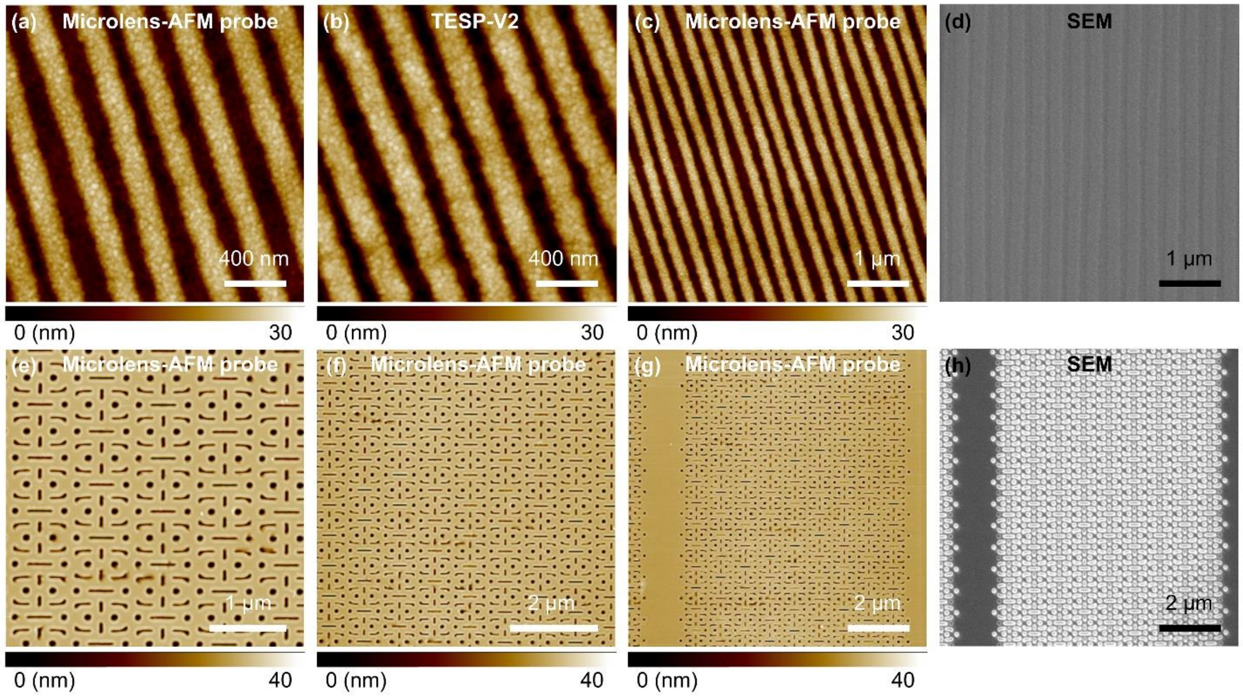

Figure S3. Experimental AFM imaging results of the custom microlens-AFM probe. (a) AFM image of a BD surface with microlens-AFM probe using tapping scanning mode. (b) AFM image of a BD sample surface with commercial AFM probe. (c) Large-area AFM scanning imaging of the BD sample using microlens-AFM probe. (d) SEM image of the BD sample surface. (e−f) AFM image of a semiconductor wafer surface with microlens-AFM probe. (h) SEM image of the semiconductor wafer surface.

Figure S3 shows the results of AFM imaging of the 210 nm interval 110 nm stripe structure of the Blu-ray Disk (BD) surface and the semiconductor wafer surface pattern with a minimum feature size of 50 nm by using a custom microlens-AFM probe. Images of the samples were taken in tapping mode with a horizontal scan rate of 0.5 Hz. The results show that the AFM imaging range is 2 μm, 4 μm, 5 μm, 7μm and 10 μm the imaging effect of AFM is comparable to that of commercial probes.

Figure S4 shows large-area AFM scanning imaging of a semiconductor wafer surface using microlens-AFM probe with a horizontal scan rate of 0.3 Hz and 512 scanning lines, the imaging areas is 60 × 60 μm^2 and 90 × 90 μm^2 respectively.

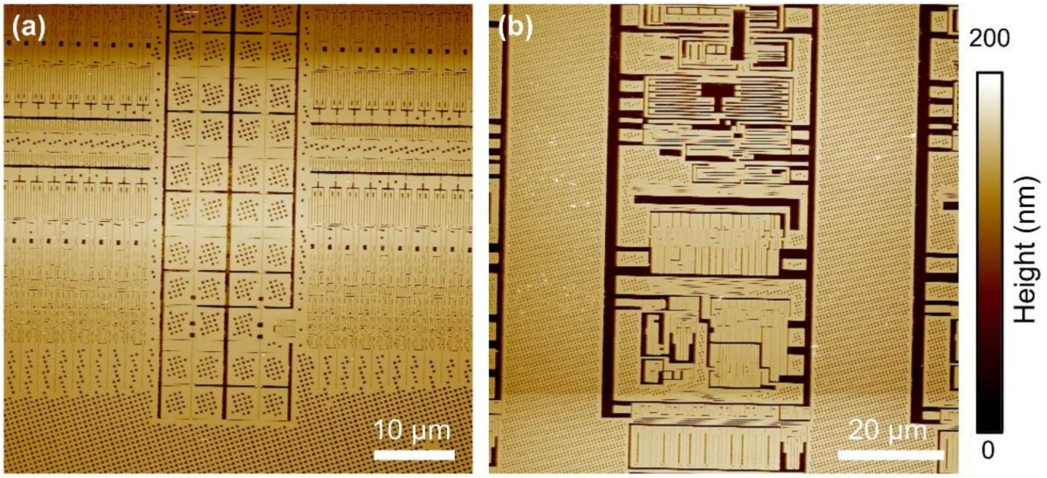

Figure S4. Large-area AFM scanning imaging of a semiconductor wafer surface using microlens-AFM probe.

Durability analysis of the custom microlens-AFM probe

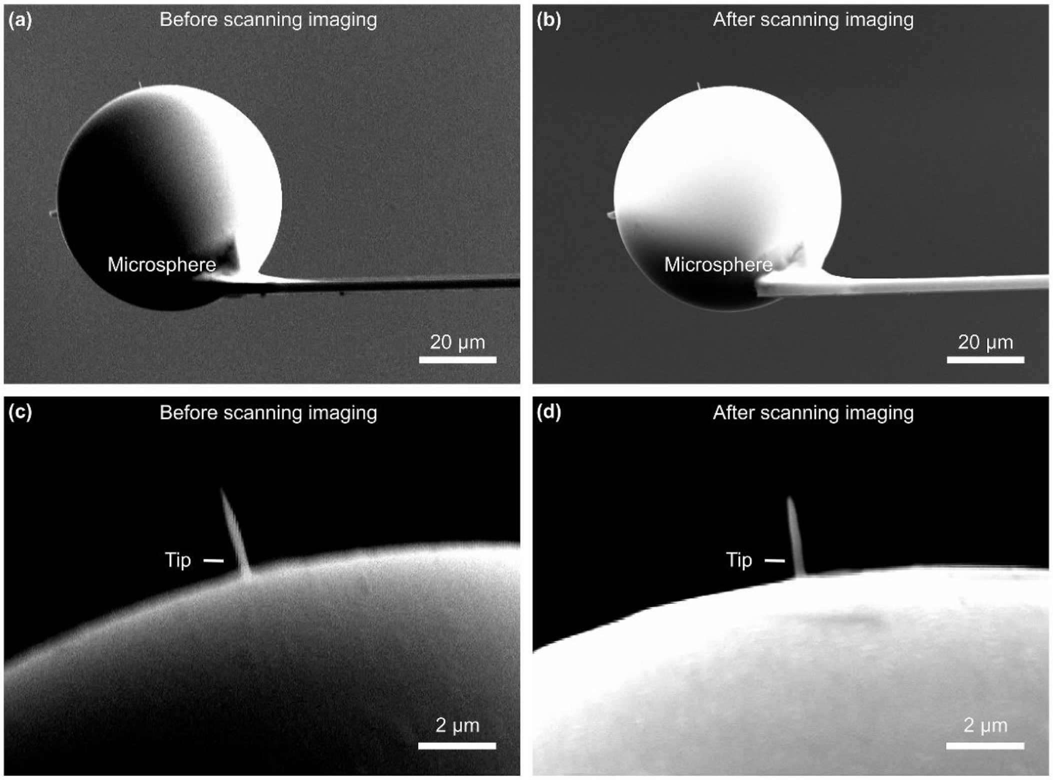

Figure S5. Durability analysis of the custom microlens-AFM probe. (a) SEM image of the microlens with a diameter of 60 μm combined at the end of the AFM cantilever and the probe tip is deposited on the microlens. (b) SEM image of the custom microlens-AFM probe after 6 hours of scanning maging. (c) Expanded view of (a). (d) Expanded view of (b).

The SEM image the custom microlens-AFM probe is shown in Figure S5a, the diameter of the microlens is 57 μm, the length of deposition tip is 2.05 μm. After 6 hours of scanning imaging, SEM examination of the tip revealed no appreciable change in the tip. And the test results show that, one probe can be reused more than 10 times on average.

Optical imaging and performance analysis of the scanning microlens

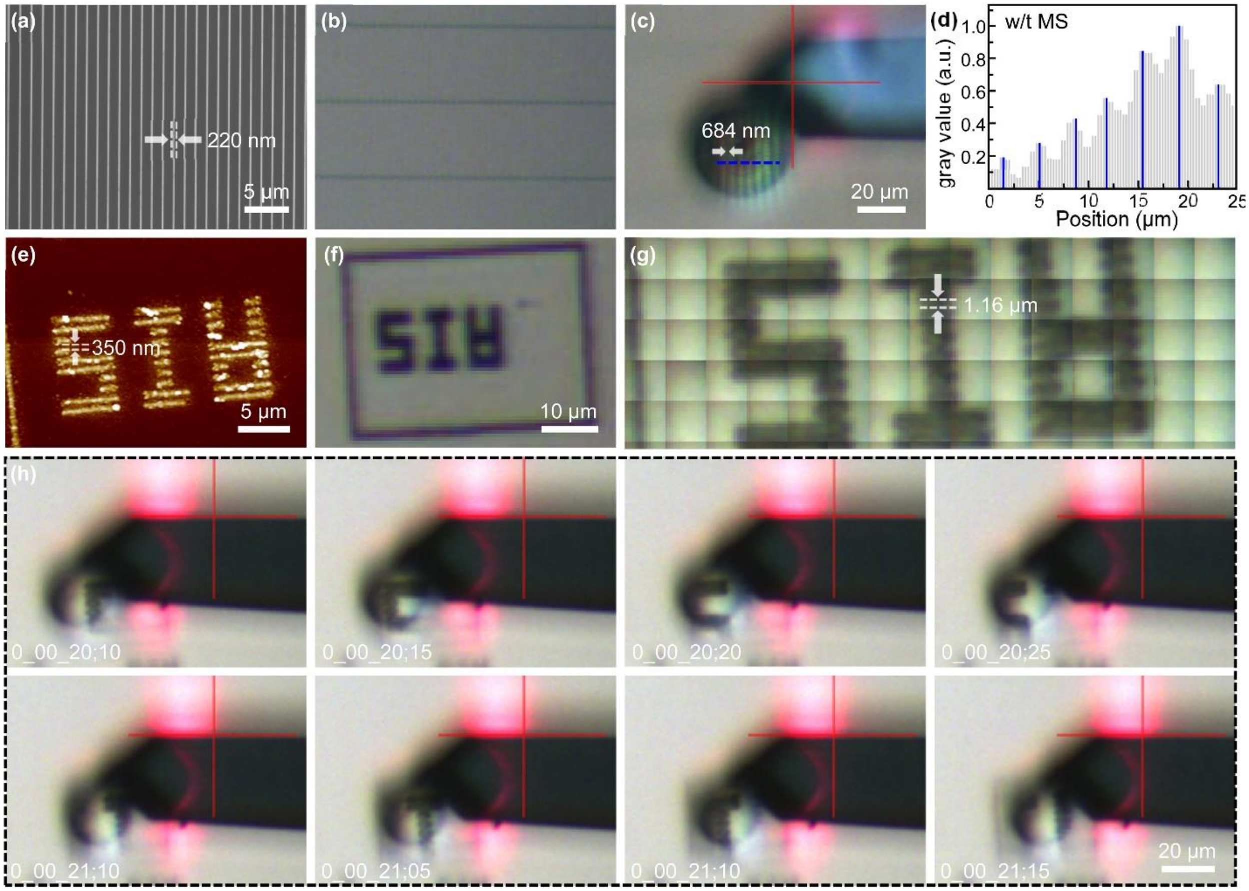

Figure S6. Optical imaging and magnification factors analysis of the scanning microlens. (a) SEM image of the stripe structure 220-nm-wide lines separated by 1 μm on a semiconductor wafer. (b) Image directly observed using the attachment optical system of AFM. (c) Stripe structure imaged using scanning superlens microscopy. (d) Normalized gray values along the blue line in the scanning superlens microscopy image (c). (e) AFM image of an 'SIA' pattern. (f) Image 'SIA' pattern directly observed using the attachment optical system of AFM. (g) 'SIA' pattern imaged using scanning superlens microscopy. (h) The process of scanning imaging in (g).

In order to verify the optical imaging and performance of the microlens, the stripe structure 220-nm-wide lines separated by 1 μm on a semiconductor wafer (Figure S6a) which could not be discerned by the attachment optical system of AFM (Figure S6b). Optical microscope image of

the stripe structure with the enhancement of the microlens with 43 μm diameter is shown in Figure S6c. With assistance of the microlens, the width of the stripe was magnified from 220 to 684 nm with magnification factors of 3.11×. As shown in Figure S6e 'SIA' pattern processed by femtosecond laser were used as samples, the fine detail of the letter is well resolved with magnification factors of 3.32×when using the scanning microlens with a diameter of 30 μm. The imaging area of 12 × 25 μm^2 can be obtained by scanning and stitching (Figure S6g). The process of scanning imaging is shown in Figure S6g, the imaging time is less than 1 minute.

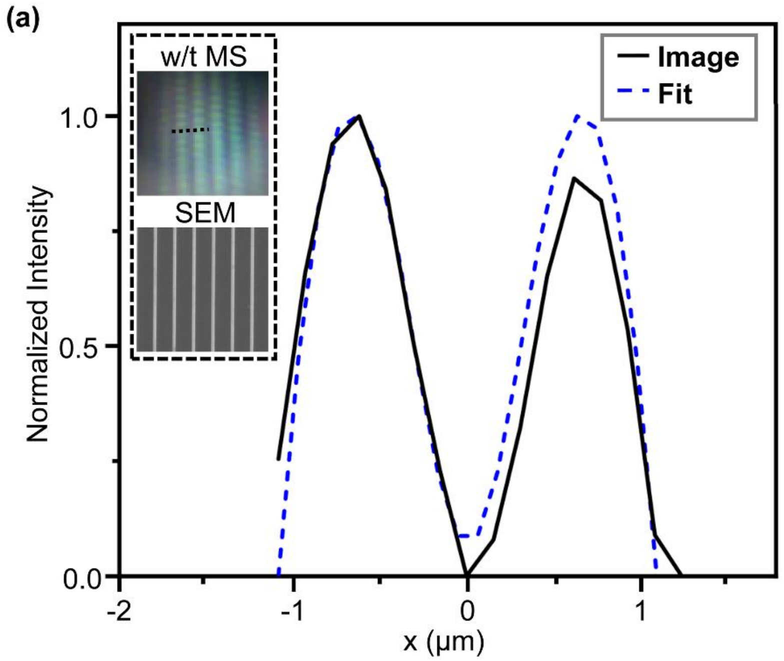

Figure S7. Optical imaging and resolution estimation of the scanning microlens.

From the surface structure data of the semiconductor wafer sample measured by SEM, we idealized the sample information to construct a striped intensity distribution function with 0.22-μm-wide lines separated by 1 μm. Convoluted it with the Gaussian fitting point spread function (PSF), and matched with the experimental data observed by the microlens. Through multiple iterations, the PSF and FWHM of the system are evaluated. With this method, we estimated that the imaging resolution of the scanning microlens system is approximately 550 nm with the silica microsphere (diameter ~ 40 μm).

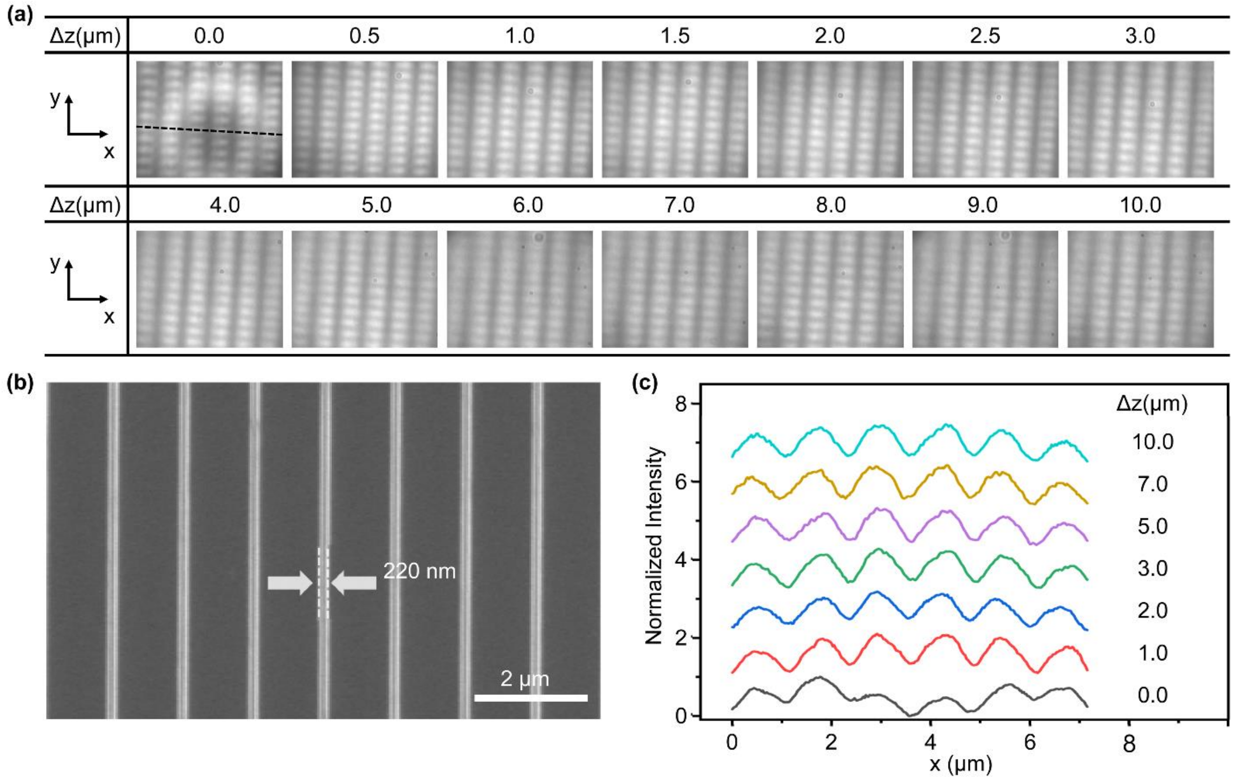

Figure S8. The influence of the distance between microlens and sample (tip length) on optical imaging. (a) The clearest optical image of the scanning microlens observed at different Δz. (b) SEM image of the stripe structure 220-nm-wide lines separated by 1 μm on a semiconductor wafer. (c) The normalized intensity of contour information extracted at the position marked in (a).

In order to further analyze the imaging characteristics of the microlens and study the influence of the distance between the microlens and the sample (Δz) on the imaging performance, the clearest images observed by the microlens at different Δz were recorded, as shown in figure S8a. When Δz = 0, that is, the microlens contacts the sample, Newton's ring appears in the imaging results due to the interference effect. From the standardized results of the contour information extracted from these results, it can be seen that when Δz is less than 10 μm, the microlens can maintain the imaging quality of the semiconductor wafer sample with the stripe structure 220-nm-wide lines separated by 1 μm, and the distance has little effect on the results (figure S8c). Therefore, we speculate that in the tapping mode, the deposition tip height of the custom microlens-AFM probes is less than 10 μm, which can ensure the optical imaging of the microlens within the appropriate working distance.

Microlens-AFM simultaneous imaging

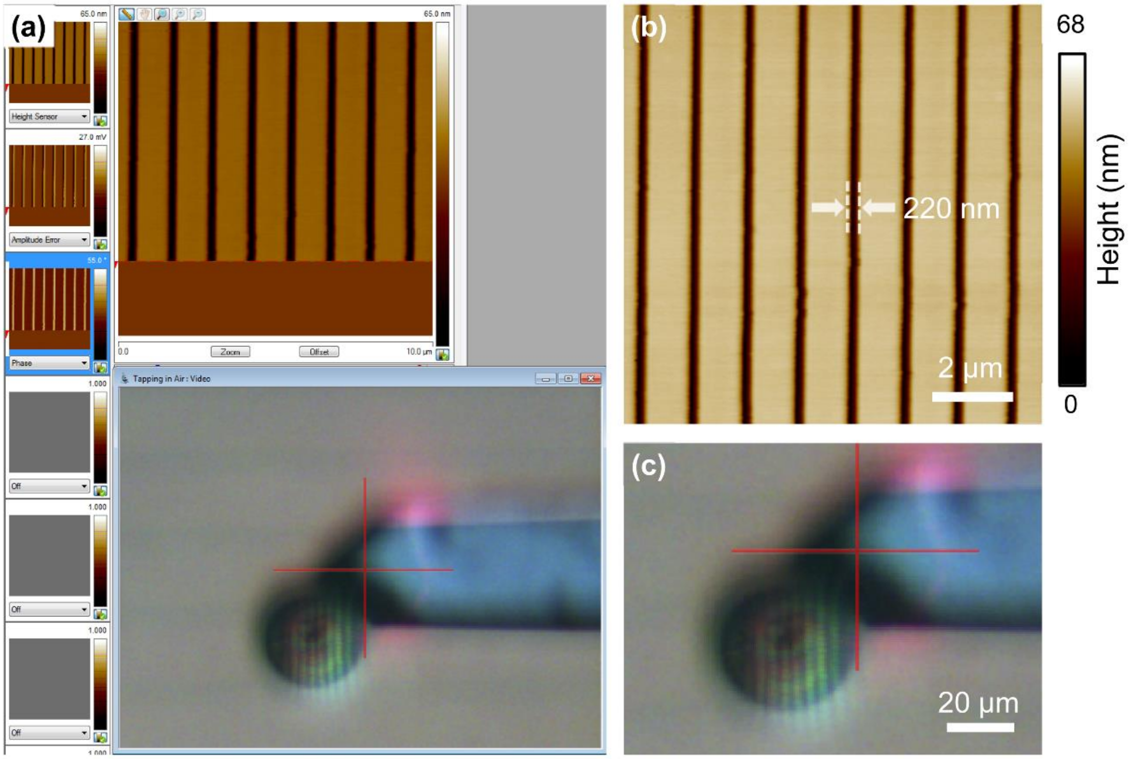

Figure S9. Microlens-AFM simultaneous imaging. (a) Interface of microlens and AFM scanning imaging simultaneously. (b) AFM image of the stripe structure 220-nm-wide lines separated by 1 µm on a semiconductor wafer. (c) Optical imaging with the enhancement of microlens.

Figure S9 shows the imaging of stripe structure of Semiconductor wafer surface in microlens-AFM simultaneous imaging modes using correlated AFM and scanning superlens microscopy, the interface of microlens and AFM scanning imaging simultaneously is shown in Figure S9a. The sample surface was scanned in air at a scan rate of 0.2 Hz, by a customized microlens probe with a silica 43.1-µm diameter microlens and a diamond material tip of 1.8 µm in length. Figure S9b shows the AFM imaging results of the sample surface though 256 lines scanning, with a total scanning area of 10 × 10 μm^2. At the same time, the optical images recorded by the scanning superlens microscopy (Figure S9b). Supporting Information movie S1 for the dynamic real-time video recorded during the imaging process.
